# Alimentation in Disease

**Published:** 1868-06

**Authors:** 


### Alimentation in Disease.

The *Lancet* thus speaks of Dr. Flint's article on "Alimentation in Disease," in the February number of this Journal:

"Among American physicians there are few who speak with more authority than Dr. Austin Flint. The February number of the *New York Medical Journal* contains an admirable paper by him on the subject of 'Alimentation in Disease,' read at a recent meeting of the Medical Society of the county of New York. We regret that we can only give an idea of the drift of this paper. Beginning with a eulogium on Chomel's definition of the art and practice of medicine as the application of good sense to the treatment of diseases, he proceeds to a personal vindication of that excellent, if not 'common' quality, and thereafter to his subject. It is painful to think how many errors scientific men would have been kept from if they had given a little more play to their own good sense. On the subject of alimentation Dr. Flint is in practical accord with those physicians who have lately insisted on the importance of nourishment in the treatment of disease. After briefly summarising the natural history of starvation, he goes on to show that the phenomena of starvation are not confined to cases in which there is a complete deprivation of aliment—in other words, that there are all degrees of innutrition—that degrees of it may be produced in diseased persons as well as in healthy ones, 'that starvation is sure to occur in cases of disease in a degree proportionate to the lack of material for nutrition in the blood,' with the same effects and phenomena which attend starvation in persons in previous health. Further, Dr. Flint shows that this starvation may supercede the disease, and kill the patient when the disease itself would not do so, or kill him sooner than if the effects of starvation had not been added to those of disease. Dr. Flint treats of the question of limitations of nourishment. He believes that, excepting perhaps in the early stage of acute disease, there is never any risk of hyper-nutrition, and thinks it 'always desirable to supply aliment to the fullest extent of the capacity of the organism for appropriation.' In both acute and chronic disease he recognizes the importance of nourishment. 'In acute diseases the failure of the vital powers is forestalled in proportion as nutritive supplies are assimilated.' In chronic diseases, 'no matter what may be the seat or nature of the chronic affection, a diet fully up to the capacity of the organism for nutrition promotes recovery, if recovery be possible; and if recovery be not possible, by increasing the ability of the system to endure the affection, contributes to prolong life.' It is, of course, desirable to avoid over-alimentation; but this can easily be done, and, at the worst, the evils of it have been exaggerated. As practical rules for avoiding on the one hand over-alimentation, and on the other disorder of digestive organs, he suggests that alimentation must often be regulated without regard to indications afforded by appetite or taste. He advocates the allowance of a sufficient period for digestion and for rest, pleasant changes of food, great consideration for the peculiar cravings of patients as representing generally a want of nutrition. He opposes many popular errors on the subject, and speaks happily of the pampered idiosyncrasies of many persons on the subject of diet as a 'strange manifestation of egotism.' We have said enough to show that this paper contains a great amount of wisdom on an important branch of therapeutics, expressed in a happy and aphoristic style. Probably Trübner & Co. could supply our readers with the Journal which contains the article, which is one well worth both reading and keeping."—*New York Journal.*

---

Dr. John Davy, brother of Sir Humphrey, died lately at the age of 78. Until a short time before his death he held the post of surgeon in the British army, and to the last he was engaged in important chemical researches.

